# Subjective Health Status and Unmet Medical Needs in Older Adults With Hypertension

**DOI:** 10.1093/geroni/igaf122.3400

**Published:** 2025-12-31

**Authors:** Seolah Yoon, Min Kyung Park, Dahye Hong, Sikyeong Park, Bada Kang

**Affiliations:** Yonsei University College of Nursing, Seoul, Republic of Korea; Yonsei University College of Nursing, Seoul, Republic of Korea; Yonsei University College of Nursing, Seoul, Republic of Korea; Yonsei University, Seoul, Republic of Korea; Yonsei University College of Nursing, Seoul, Republic of Korea

## Abstract

Hypertension is a common chronic disease among older adults that necessitates regular medical care. However, when individuals need medical care but cannot access it, they experience unmet medical needs (UMN). Poor subjective health status and depression may exacerbate this situation by discouraging healthcare-seeking behaviors; however, few studies have explored this relationship. This study examined the association between subjective health status and UMN and the moderating role of depression using data from the Korea Community Health Survey (KCHS). A secondary data analysis was conducted on 44,400 individuals aged 65 and older diagnosed with hypertension. A logistic regression model was used to assess the relationship between subjective health status and UMN, incorporating depression as a moderating factor, while adjusting for covariates such as age, sex, stress level, education, and economic activity. Among older adults with hypertension, 15.6% experienced depression, and 5.0% reported UMN. Individuals with poorer subjective health were 46% more likely to experience UMN than those with better health perceptions (Odds Ratio [OR] = 1.46, 95% CI: 1.36–1.56, p < 0.001). However, among those with depression, the impact of subjective health deterioration on UMN was 11% lower (OR = 0.89, 95% CI: 0.78–0.98, p = 0.048). Future research should further categorize UMN and examine the mechanisms linking subjective health status and depression to UMN. These findings can inform targeted interventions to address UMN in aging populations, emphasizing the importance of addressing psychological barriers to healthcare access.

